# Perception of healthcare workers on mobile app-based clinical guideline for the detection and treatment of mental health problems in primary care: a qualitative study in Nepal

**DOI:** 10.1186/s12911-021-01386-0

**Published:** 2021-01-19

**Authors:** P. Pokhrel, R. Karmacharya, T. Taylor Salisbury, K. Carswell, B. A. Kohrt, M. J. D. Jordans, H. Lempp, G. Thornicroft, N. P. Luitel

**Affiliations:** 1 Research Department, Transcultural Psychosocial Organization (TPO) Nepal, Kathmandu, Nepal; 2 Centre for Global Mental Health, Health Service and Population Research Department, Institute of Psychiatry, Psychology and Neuroscience, King’s College London, London, UK; 3 Department of Mental Health and Substance Use, World Health Organization, Geneva, Switzerland; 4 Department of Psychiatry, George Washington University, Washington, DC USA; 5 Centre for Rheumatic Diseases, Department of Inflammation Biology, School of Immunology and Microbial Sciences, Faculty of Life Sciences and Medicine, King’s College London, London, UK; 6 Centre for Global Mental Health and Centre for Implementation Science, Health Service and Population Research Department, Institute of Psychiatry, Psychology and Neuroscience, King’s College London, London, UK

**Keywords:** Mental health, mhGAP, Mobile technology, Primary care, m-health, Nepal, Qualitative study

## Abstract

**Background:**

In recent years, a significant change has taken place in the health care delivery systems due to the availability of smartphones and mobile software applications. The use of mobile technology can help to reduce a number of barriers for mental health care such as providers’ workload, lack of qualified personnel, geographical and attitudinal barriers to seek treatment. This study assessed the perception of Nepali primary healthcare workers about the feasibility, acceptability, and benefits of using a mobile app-based clinical guideline for mental health care.

**Method:**

A qualitative study was conducted in two districts Chitwan and Ramechhap of Nepal with purposively selected medical officers (n = 8) and prescribing primary healthcare workers (n = 35) who were trained in the World Health Organization mental health Gap Action Program Intervention Guide. Semi-structured interviews and focus group discussions were conducted in Nepali, audio recorded, transcribed and translated into English for data analysis. Data were analysed manually using a thematic analysis approach.

**Results:**

The majority of the healthcare workers and medical officers reported a high level of interest, motivation and positive attitudes towards the mobile app-based clinical guidelines for detection and treatment of people with mental disorders in primary care. They respondents suggested that several features and functions should be included in the app: suggestive diagnosis and treatment options; clinical data recording system; sending messages to patients to promote follow-up visits; allow offline functions; minimal typing options and content to be available in Nepali language. The study participants reported that the app could help in bringing uniformity in diagnosis and management of mental disorders across all health facilities, enabling remote supervision, helping verification of health workers’ diagnosis and treatment; and increasing patients’ trust in the treatment. Lack of reliable internet connection in health facilities, possibility of distracting interaction between patient and provider, and confidentiality were the key factors potentially hindering the use of the app.

**Conclusion:**

The suggested functions and features as well as the potential risk factors highlighted by the health workers, will be considered when further developing the mobile app-based clinical guidelines, training modality and materials, and the supervision system.

## Background

Despite the availability of effective treatments for mental illness, there is a huge gap between the number of individuals who need mental health care, and those who receive treatment. The recent World Health Organization (WHO) World Mental Health Survey initiative reported that 86.3% of people with anxiety, mood or substance disorders in low or middle-income countries (LMICs) received no treatment in the past 12 months [1]. Among people who do receive treatment, many receive inadequate treatment [2]. For example, a recent study conducted in 21 countries found that only 1-in-27 people living with major depressive disorder in LMICs receives minimally adequate treatment [3]. The most common barriers for mental health care utilization include low perceived needs, stigma and discrimination associated with mental illness, inability to afford the treatment cost, poor identification and referral system, and shortage of human resources [4–10].

Over the past decade, several initiatives have been undertaken globally to minimize the treatment gap for people with mental disorders. One of the most widely used approaches is the task-sharing, which involves specialist mental health workers (e.g. psychiatrists and psychologists) in designing and managing mental health services, building clinical capacity of primary and community health care workers to provide direct mental health care services with regular supervision and quality control [11, 12]. In 2008, WHO launched the mental health Gap Action Programme (mhGAP) [13], and the Intervention Guides (IG) in 2010, with the aim of providing evidence-based guidance to primary health care workers for detection and treatment of MNS disorders in primary care [14]. The mhGAP-IG has been found to be effective in offering basic mental health services through primary health care systems [15, 16]. However, due to the scarcity of mental health specialists in many LMICs, this approach alone may not be adequate to close the substantial mental health treatment gap [17].

In the recent years, a significant change has taken place in health care delivery system due to the availability of smartphones and smartphone applications [18]. The use of mobile technology can help to reduce a number of barriers for mental health care such as providers’ workload, lack of qualified personnel, geographical and attitudinal barriers to seek treatment [19]. The use of mobile devices in healthcare (also called m-Health) consists of interventions that use mobile phones and other digital devices (e.g. smartphones, tablets) for health care purposes that includes: (a) creating awareness about mental health problems; (b) screening and diagnosis of mental disorders; (c) supporting clinical care; (d) training and supervision of non-specialists; and (e) improving treatment adherence [20, 21]. In 2017, the WHO launched an electronic version (e-version) of the mhGAP-IG v2.0 to facilitate the use of technology in mental health care [22]. Studies conducted in high income settings demonstrated that mobile technologies can be applied effectively in clinical decision making by health care professionals [23]. These technologies have not yet been used widely in LMICs for mental health care delivery; however, the available data demonstrates that mobile app-based clinical guidelines help to improve protocol compliance [24–26] and can facilitate task-shifting, and increase health workers morale [25, 27]. Despite these benefits, there are many potential barriers to implement m-Health solution, including suitability and acceptability to both healthcare worker and service user [26]. Developing a mobile app-based clinical guide using Human Centered Design (HCD, or user-centered design) may result in more engaging interventions as HCD actively include concerned stakeholders in the process of intervention development using cutting-edge methods to optimize for end-users and provide a solution for key problems that are being addressed [28].

In Nepal, the number of mobile device contracts (i.e. 27.85 million in 2020) is more than the total population of the country (i.e. 26.49 million) [29]. However, few studies have assessed the feasibility and effectiveness of the application of mobile technology in health care delivery within the country. These studies have attempted to test if mobile devices can support maternal and new born child healthcare, particularly for patient data collection to send reminders for follow-up and assess signs and symptoms of illness [30]; gather health surveillance data from rural areas [31]; SMS reporting of neonatal health information and malaria surveillance [32]; support health workers in the rural district hospitals, including provision of remote specialists services [33]; aiding community health volunteers to engage persons with mental illness in treatment initiation at primary care facilities [34]; and remote supervision for mental health services [35]. None of these studies has attempted to assess the feasibility of an app-based decision aid for mental health diagnoses in primary care.

This study was conducted as a part of the **E**-**m**hGAP **I**ntervention guide in **L**ow and m**i**ddle-income countries: proof-of-concept for Impact and **A**cceptability (Emilia) project, which aims to determine the feasibility of implementation of an adapted e-version of the mhGAP-IG in primary care in Nepal and Nigeria. This study builds on our previous work in Nepal where the mhGAP-IG was adapted, evaluated and scaled-up [36, 37]. Understanding of the end users’ (i.e. primary healthcare workers) interest, motivation and concerns on a mobile app-based clinical guide is important to support the development of the app and its implementation strategy. Therefore, this study assessed the perception of mhGAP-IG trained primary health care workers on feasibility, acceptability and benefits of using mobile app-based clinical guideline for detection and treatment of mental health problems in primary care setting, and the possible features and functions to be included in the mobile app.

## Methods

### Setting

The study was conducted in Chitwan and Ramechhap districts in the Bagmati province in central Nepal. Ramechhap district lies in the hilly region, which has total population of 202,646 with a population density of 131 per sq. km. Similarly, Chitwan district lies in the Terai region which has total population of 579,984 with a population density of 261 per sq.km. Chitwan has comparatively better literacy indicators than Ramechhap (76.98% in Chitwan and 62% in Ramechhap), and per capita income (Chitwan-$1537 and Ramechhap-$951) whereas life expectancy in Ramechap is higher Chitwan (Chitwan-69.78, and Ramechhap-72.90) [38]. The majority of primary healthcare facilities in Chitwan have good internet connectivity and mobile networks. There is a psychiatric outpatient department in the government hospital and two private medical colleges in Chitwan. In Ramechhap, internet connection is available only in a few primary healthcare facilities. The mobile network is also poor. No specialist mental health service is available in Ramechhap district. In Chitwan, all primary healthcare workers were trained on mhGAP-IG by the Transcultural Psychosocial Organization (TPO) Nepal through the PRIME project, which aimed to generate new evidence on implementation and scale-up of mental health services in primary care [39]. In Ramechhap, 46 primary healthcare workers from 23 health facilities (out of 56 of two Municipalities (i.e. Manthali and Khadadevi) were trained as part of TPO Nepal’s post-earthquake psychosocial support programme [40].

### Study design

The study used a qualitative study design because this method helps to explore detailed information about why a primary healthcare worker might be interested or opposed in using technology, and facilitating factors and possible barriers when using an app-based clinical guideline in assessment and management of mental health problems in primary care. The data gathering included semi-structured Interviews (SSI) and Focus Group Discussions (FGDs) [41] with primary healthcare workers who were trained in the WHO mhGAP-IG.

### Recruitment

The participants were purposively selected [42] to meet the pre-defined criteria: (a) primary healthcare workers working in Chitwan and Ramechhap districts (b) be medical officer (MO) or health assistant (HA), or senior auxiliary health worker (S-AHW) or auxiliary health worker (AHW) (c) trained on mhGAP-IG (d) currently engaged in mental health service delivery and (e) be able to provide informed consent for the study. In total, 28 SSIs (6 in Ramechhap and 22 in Chitwan) and 2 FGDs (n = 15) were conducted in Chitwan. Of the total 28 SSIs, 4 were conducted with medical officers, 15 with HAs, 6 with S-AHWs and 3 with AHWs, while both FGDs were conducted with Medical Officers, HAs and Sr-AHWs/AHWs together.

### Interview topic guide

The semi-structured interview guides were used for data collection incorporating the human-centered design principles [28] with a strong focus not just on questions of preferences, but also provide examples of actual use of digital solutions to explore some of the critical assumptions underlying the project (e.g. potential use of a m-Health solution, availability of internet). The interview guides included a range of topics such as: (1) health workers’ interest, attitudes and concerns in using technology in clinical setting; (2) health workers’ familiarity with technology; (3) experience of using technology in health care; (4) feasibility; (5) acceptability of using mobile app-based clinical guideline in the primary care (e.g. other existing app or programs); (6) perceived benefits of using a mobile app-based clinical guideline in assessment and management of mental health problems; (7) features and functions to be included in the app; and (8) potential barriers and risk factors for using a mobile app-based clinical guideline use to support decision making in routine healthcare setting, particularly in mental health care. The interview guides were pilot-tested with two primary healthcare workers in Chitwan prior to the main study. With the exceptions of language editing and question order, there was no other major revision for the interview after the pilot-test.

### Interview process

The study was conducted between February and March 2019.Written and oral information about the objectives and process of the study was provided to each study participants before their enrolment in the study. Participants provided a written informed consent to confirm their participation in the study. Two qualified and experienced researchers carried out the interviews and FGDs. Both SSIs and FGDs were conducted in a private room within the primary healthcare facility in Nepali language. One of the researchers facilitated the interviews and FGDs while the other researcher ensured correct audio recording of the sessions and took field notes. On average, the SSIs lasted for approximately 60 min while FGDs took 90 min.

### Data analysis

The audio-recorded SSIs and FGDs were first transcribed in the original language, (Nepali) by the interviewers immediately after the field work. The transcriptions were then translated into English for analysis. The data were analyzed thematically to identify and generate patterns of codes and emerging themes from the data [43]. An inductive analysis was carried out to allow unexpected themes to be identified. The transcripts were all printed and thoroughly read by two researchers independently to familiarize themselves with the data, and generate initial codes. The codes identified by each researcher were discussed further with other members of the research team and the differences in the codes of two researchers were finalized in consensus. The interviews were re-read line by line and were color coded to assign the content to the particular themes and then organized within the identified themes. Similarities and differences within the data were grouped and organized in sequence. Finally, the analysis and interpretation of the data were conducted by the same two researchers together.

## Results

### Characteristics of the participants

Forty-three health workers (SSIs: 28 and FGDs: 15) participated in the study with the mean age of 36.3 years (range 28–45 years). Among them, 31 (72%) were male, 27 (63%) had experience using any m-Health approach in their clinical work, and two (4.65%) health care workers did not own a smartphone. The mean number of years of experience in mental health was 2.02 years (range 1.25–5.29 years).

The qualitative data analysis generated an analytical framework with 5 key themes: (1) facilitating factors of using the app based clinical guideline; (2) perceived barriers for using the app based guideline; (3) perceived benefits of using the app-based guideline; (4) perceived risk factors of using the app in clinical practice; and (5) suggested functions and features by the health workers (Table 1).

**Table 1:** Summary of the results

Themes	Key findings
(1) Facilitating factors for using the app	Availability of internet in most of the health facilities Familiarity with smart phones and tablets Experienced in using m-health apps Experienced in online recording and reporting system
(2) Perceived benefits of using the app	Guides and supports in diagnosis and treatment Verifies health workers' diagnosis Easily accessible Brings standardization of diagnosis and management among all health workers and health facilities Makes remote supervision possible May increase patients’ trust in treatment
(3) Perceived barriers for using the app	Lack of internet in some of the remote health facilities Limited experience with digital technology among senior health workers Limited use of typing feature by health workers in mobile phones Concern that patients may doubt health workers’ skills and competencies
(4) Perceived risk factors of using the app	May distract from the interaction with patient Adds an extra task to enter the same information in the app as well as in patient register until government adapt online reporting system May take more time to enter information, so may not be possible to use app during busy clinics May make patients experiencing delusions more suspicious Confidentiality of patients’ data Less chance of using the app continuously when health workers becomes familiar with all aspect of the app
(5) Suggested features and functions in the app	Suggestive diagnosis and treatment after entering the symptoms of a particular disorder Minimal typing function Inclusion of patient record system in the app Send reminder messages to the patients about the follow-up appointments Content in both English and Nepali languages Short and simple sentences with large font size Offline function Inclusion of other relevant reference material (e.g. videos of psychosocial support, deep breathing exercise) in the app

### Facilitating factors for using app based guideline in clinical practice

Several facilitating factors that enable the use of the app based clinical guideline were reported in the interviews and FGDs. These included health workers’ familiarity with smart phones and other technologies in health care, and availability of reliable internet connections in the health facility. Most (n = 41) health workers owned a smart phone for phone calls, browse the internet for entertainment and to search for information required for clinical case management whenever they experienced confusion (e.g. verifying diagnosis, treatment, medicine dosage, and side-effects).

“I use mobile to make calls, use YouTube and Medscape app [a clinical decision support app] to see medication’s dose and when I have any confusion regarding the diagnosis”. **Medical Officer**

Due to the provision of online recording and reporting of the routine data such as Health Management Information System (HMIS), Electronic Immunization Reporting System (EIRS), and the Integrated Management of Newborn and Childhood Illness (IMNCI) through mobile app, internet connectivity was available in most of the health facilities, particularly in Chitwan district. Several health workers (n = 27) also reported that they use clinical decision support apps (e.g. UpToDate and Medscape) when they require information during clinical practice.

“If we [health workers] have [an] app, it will be easy because nowadays we have internet facility and therefore, it will be easier to work by looking at the mobile phones rather than to turn to a book. If we use mobile phones, all information can be found there”. **Health Assistant**

Moreover, most of the health workers (n = 41) were familiar and experienced in using Wi-Fi or mobile data to access online reporting systems; app based clinical management guidelines, search information online, and social networking and communication-based apps.

“We [health care workers] use UpToDate and Medscape app mostly. Those apps can be used offline as well to see the drugs’ dose and medical procedures. Similarly, we can also connect with other [health worker] and share our [medical] problems to get the solution on that [problem]. I mostly use it [apps] during office time and also when I am free [not working]. **Medical Officer**

### Perceived benefits of using the app based clinical guideline

A commonly reported benefit of the mobile app-based clinical guide was about its accessibility and ease of use. Health workers pointed (n = 7) out that having the app in the mobile phone makes it accessible from anywhere and anytime. This would remove the need to carry a cumbersome paper version of the guidance which could be easily misplaced.

“It is difficult to keep the book in my pocket whereas; the app can be in my pocket. Whenever I am confused, I can easily use it [the app] in my phone”. **Medical Officer**

Another frequently reported benefit of the mobile app-based clinical guidelines was standardized diagnosis and management across all health workers and health facilities. Several participants (n = 4) also articulated that because the general public is also exposed to the latest technology, when used for treatment some patients may trust the treatment provided to them, which in turn may increase the chance of treatment adherence.

“I think the most important benefit of an app [app based clinical guideline] could be in bringing the uniformity in the diagnosis among all healthcare workers”. **Health Assistant**

“I use IMNCI [Integrated Management of Childhood Illness] app in front of patient to confirm diagnosis and treatment otherwise; I use it [app] after completing patient consultation and lab investigation report.” **Medical officer**

Some health workers (n = 4) stated that they would not need to memorize the protocols if included in the mobile phone app. They emphasized that there are many guidelines and protocols that are used in the clinical practice in general health care, and are difficult to remember if they are not used for a long time. In such cases the mobile phone app was reported as helpful tool, particularly in the clinical settings.

Some participants (n = 3) also reported that using the app in front of the patient was more comfortable than using a book.

We [health workers] cannot remember everything given in the protocol; therefore, we need to use books/manuals during the consultation. However, it is difficult to use manuals or books in front of the patients because patients think that the health worker is not good if he/she uses books or manuals during consultation. **Senior Auxiliary Health Worker**

Another benefit reported by the health care workers was to accessing patients’ data whenever they are needed. Health workers reported that many patients forget to bring their Out Patient Department (OPD) card (i.e. clinical notes) in the follow-up visits, or sometimes even lose their medical reports; app might be very helpful to access patients’ medical records.

“App [e-mhGAP] is good for recording of patient information as patient may lose or forget the prescription slip when coming for follow up. So, even when they [patients] do not bring the prescription slip, we [health workers] will be able to see all the information of the patient in the app and provide service as needed”. **Health Assistant**

Finally, health workers also considered the app-based clinical guide as a means to improve and update their mental health knowledge. They reported that they will go through the app, particularly, the reading materials included in the app whenever they have free time. Such an instant accessible resource is helpful to enhance their mental health knowledge.

Whenever I want to learn and refresh my knowledge; I can use the app [e-mhGAP]. Even if a supervisor is not assigned to me, or refresher training is not provided, the app [e-mhGAP] can help me revise whatever I learned in the training. **Medical Officer**

### Perceived barriers of using the app based clinical guideline

Lack of internet connection in the remote health facilities emerged as a major barrier for the use of mobile app-based clinical guides. Some health facilities in Chitwan, and most of the health facilities in Ramechhap districts are located in remote hilly regions where the internet connections, as well as the mobile connectivity are very poor.

“It [computer] was used for reporting of immunization data and was provided by WHO Nepal office. Nowadays, there is no internet connection in this health facility. Municipality don’t provide budget for internet [infrastructure]. A computer is just installed in health facility but has not been used”. **Health Assistant**

The type of health worker was also reported as one of the barriers that could affect the use of the mobile app-based clinical guide. For example, the majority of the participants shared that some older (aged) health workers may have limited experiences with smart phones or tablets, and therefore they may find it difficult to operate the mobile app based clinical guidelines.

“Older health workers, who are less experienced in using smart phones, might not feel comfortable to use the app, but the newly recruited health workers will use the app, even by searching about it [in the internet] by themselves**”. Health Assistant**

A few participants (n = 5) also reported that the app might be much easier and appropriate for medical officers in comparison to paramedics (a healthcare worker who has undertaken 18 months to 3 years training) because of the higher level of education of medical officers. They further stated that medical officers are experienced in using technology in their pre-service training as well as in the clinical practices. These results were also validated by our observations during the data collection, where medical officers found the e-mhGAP prototype easy to use whereas the paramedics required lot of support and guidance.

“Electronic devices are used by everyone; therefore, the app will be feasible and acceptable. However, the app could be more relevant and appropriate for medical officers because of their higher level of education compared to the paramedics”. **Medical Officer**

Another possible barrier reported by the health workers in using the mobile app-based clinical guide was about patients mistrust towards health workers’ skills and competencies to provide services. Health workers reported that using an app or book during a patient consultation is not a common practice in primary care in Nepal, hence, some of them expressed their anxiety that if they use the app during a consultation, patients might think the health worker does not know anything about case management/treatment and is not capable to treat them.

"They [patients] may think that we [health worker] know nothing;[instead] we are working by using the mobile [during the consultation].” **Health Assistant**

### Perceived risk factors of using the mobile app-based clinical guideline

Despite the numerous benefits of the mobile app-based clinical guideline reported in the study, some of the health workers also pointed out few possible risk factors of using the app in the real world. The most commonly reported was about the possibility of distracting the clinical conversation of health workers and patients. They reported that patients may feel that health workers are not paying attention to their concerns if they use the app during consultation, which may obstruct rapport building: an important component in assessment and diagnosis of a mental health problem. The health workers suggested a way forward could be to inform the patients before a consultation why they are using the app.

“I think that patients will have negative perception that we [health worker] are not concentrating on them or listening to them [patients]”. **Health Assistant**

“I used to think that the patient may think that I didn’t know anything if I use the phone during consultation. But after sometime, I started explaining to the patient and their family about why I was using the phone. Now, I am comfortable using the phone and patients are also happy when I use mobile during consultation”. **Medical officer**

Another risk factor reported in the study was about entering patients data twice i.e. in both patients register and in the app. In the existing health care system, a patient is first register in the health facility, and his/her clinical information is entered into an OPD card. If a new system (i.e. a mobile app) is introduced, patients’ data may be needed to be entered in both OPD card and in the app until government adapt online reporting system. This may create extra burden among health care providers.

“Using the app will increase the health worker’s work load instead of reducing it as they have to enter the patient’s information twice, once in the health facility’s OPD register and again in the app.” **Health Assistant**

Another point mentioned in the study was about longer consultation time when using mobile app-based clinical guides. Health workers reported that entering patients’ data and clinical notes in the app during consultation might take longer time, and it also may not be possible during high number of patient clinic appointments. They also stated that due to limited consultation time, there might be a possibility of having incorrect patients data collected in the app during rushed time. Furthermore, a few health workers also put forward that when using the app in front of the patients, other patients’ record would also be visible to them, which may break the confidentiality.

“They [patients] might feel that we [health workers] are not focusing on them [patients] if we use mobile phone during consultation. Similarly, patients may also think that confidentially would be broken because they may see the names of other persons in the mobile app”. **Health Assistant**

### Suggested features and functions in the app

Health care workers have suggested several functions and features in developing the app which included suggestive diagnosis and treatment process and options, use of local language and script (Nepali), large font size, short and simple sentences, and ability of using offline mode. Almost every health worker reported that their pre-service training was taught in Nepali medium (Nepali language). They have suggested Nepali language and script in the app. As some of the remote health facilities lack reliable internet connection, they also recommended that the app need to be able to work in offline mode. Furthermore, the large font size and short sentences as these will make it easily readable and will not take up longer time for health care workers when reading the app based guideline in Outpatient Department (OPD).

“The app [e-mhGAP] should be developed in Nepali language because not all health workers understand English”. **Health Assistant**

“The app [e-mhGAP] should be able work in offline mode because not everyone who uses the app has access to internet connection all times”. **Health Assistant**

Likewise, writing/typing into a mobile phone was not commonly practiced among the health care workers. They reported that they prefer making phone calls more often even though message service are much cheaper because making phone calls is easier than sending text messages. Therefore participants proposed that the app should have fewer typing features and that pre-defined responses would be helpful to reduce the time spent entering information and maintaining the accuracy of the patients' data.

“Instead of typing the symptoms the [list of] symptoms should be already given there [in the app] so that we can choose from the list. It will be time consuming to enter all the symptoms in the app by typing.” **Health Assistant**

Most health care workers (n = 43) recommended incorporating a decision support feature in the app. They shared that they are often confident in making diagnosis for ‘straightforward cases’ of depression, but found it difficult to diagnose patients presenting with co-morbid symptoms, e.g. depression with psychotic symptoms. As a result, the step by step functionality of the original mhGAP-IG app was considered to be very helpful, especially for health care workers with limited experience in mental health case management.

“It [e-mhGAP app] should contain all the contents of the mental health register (i.e. patients registration form at the health facility). For example, we [healthcare workers] should be able to enter patients’ information and it [the app] should contain all the symptoms of [mental] diseases. Similarly, if we [healthcare workers] choose the symptoms, let it [the app] suggest the diagnosis itself, based on the [inserted] symptoms. Similarly, let it [the app] suggest the treatment as well as the medicines’ dose. Moreover, it [the app] should be developed in Nepali so that everyone can understand [the content]”. **Medical Officer**

Compatibility of the app was highlighted as another important aspect of its incorporation into working practices. As health care workers often use their personal mobile phones to access apps for work, it is imperative that apps compatible with both Android and iOS operating systems. Additionally, health care workers also had a concern about the storage space that the app will take in the phone. Due to limited storage space of some mobile devices, health workers also recommended that the app should take minimal memory space.

“The app should not use much space of the mobile phone because the apps, which consume large storage space, are not used [by health workers]. Even I deleted that app [e-mhGAP] because it took large storage space and I didn’t use it often and there was no sufficient space to download other apps”. **Health Assistant**

“I do not use IMNCI app [Integrated Management of Childhood Illness] because it is not compatible with my iPhone.” **Medical Officer**

The next frequently recommended feature and function (n = 12) to be included in the app was a messaging feature for sending reminder to a patient for follow-up care. Health workers reported that dropout rate of patients receiving mental health treatment in primary care is relatively higher than general healthcare. Therefore, health workers recommended messaging feature through the app for improving treatment adherence among patients receiving mental health services.

“It [IMNCI app] gives a ring or message to the parents’ mobile after entering the phone number and reminder message about the follow-up date is sent. It would be far better if we can do such [a feature] in this [e-mhGAP] app”. **Health Assistant**

Some of the health workers also endorsed including the pictures of Community Informants Detection Tools (CIDT), a vignette-based tool for pro-active case detection of people with priority mental disorders [44], for assessment, and videos of psychosocial intervention. They reported that the pictures and videos could be useful demonstration materials to deliver psychosocial interventions.

“I think, it would be even easier to understand if the videos about the conversation between patient and health worker and the exercise of relaxation and deep breathing would be added”. **Auxiliary Health Worker**

In addition, some of the health workers also suggested a data sharing feature for sharing patients’ data to the supervisors in order to use in the remote supervision.

“We [health workers] can make the app [e-mhGAP] in such a way that the app has a common conversation place where we would be able to upload our confusions [about case management] and our colleagues (health workers) will suggest us. Similarly, it will be more helpful if we would be able to send the information of the patient to the psychiatrist.” **Medical Officer**

For supervision, the app [e-mhGAP] should have a feature that would allow us to enter the information of the case [patient situation] and the supervisor will review it [information], and suggests required steps [for case management] to be taken. **Medical Officer**

## Discussion

This study assessed the perception of primary healthcare workers about the feasibility, acceptability, and benefits of using a mobile app-based mhGAP-IG in the detection and treatment of people with mental health disorders in primary care in Nepal. Most of the health workers who participated in the study reported high level of interest, positive attitude and motivation in using mobile app-based clinical guideline to support mental health treatment. As mobile technologies and internet facilities are increasingly expanded to the rural areas of Nepal; mobile app-based mhGAP-IG could be a viable and effective strategy in scaling -up of the primary care based mental health services throughout the country. A number of potential benefits have been reported by primary health care workers in using the app which included: (1) minimization of the barriers related to low detection of mental health disorders in primary care; (2) possibility of remote supervision of the trained health care workers; (3) increase in the treatment coverage of mental health services from primary care; (4) bringing uniformity in diagnosis and management of mental disorders across all health workers; (5) convenience and easy to use; and (6) validating health workers diagnosis and treatment process and options.

Decision support options in the detection and treatment of mental disorder was one of the most commonly recommended functions in the app by all the health workers participating in the study. A similar advantage of m-health interventions was also reported in a study conducted in Uganda [27], and in a systematic review of the feasibility and effectiveness of m-health strategies in LMICs [45]. In our recent study among the same health care workers in Chitwan, the detection rates of depression and alcohol use disorder in primary care increased significantly (i.e.15.5 points for depression and 58.9 points for AUD) after the introduction of the mhGAP-based training; however, the rates reduced substantially (i.e. 10.2 points increase for depression and 12.1 points for AUD) 2 years after the training [36]. As the correct detection of mental health disorders is an important indicators for initiating a minimally accurate treatment; therefore, using mobile app-based clinical guide could be a viable and effective strategy to improve detection, diagnosis and treatment of mental health disorders in primary healthcare.

However, use of such technology is not without barriers, as demonstrated by this study. These perceived risks and limitations highlighted the importance of a user centered approach to design of such interventions. For example, limited experience of using digital technology among older (aged) health care providers was highlighted as a potential barrier to uptake of the mobile app-based clinical guidelines. Similar challenges have previously been reported in other studies. For example, a study conducted to assess the acceptability and feasibility of using digital technology in mental health care in rural India also reported limited familiarity of health care providers’ with digital technology as a key challenge for delivering depression care [46]. Similarly, our results also support the findings of the review of available statistics and evidence of smartphone use among elderly people which demonstrated that they use a smartphone for making calls but rarely did they utilized downloaded apps [47].

A systematic review on the feasibility and effectiveness of m-Health strategies for frontline health workers in LMICs reported that if adequate training is provided to the frontline workers, they can easily learn the technology for improving the services they provide [45]. Similarly, a study conducted to assess the feasibility of using smartphones in maternal health care in Ethiopia reported that the health care providers who did not have previous exposure to smart phones and electronic devices were able to use the phones effectively in day-to-day maternal health care service delivery [48]. A pilot programme in rural Nepal which tested the feasibility of using mobile phones for collecting health surveillance data reported that community health workers can collect health surveillance data accurately using mobile technology if they are trained adequately [31]. There have also been studies in Nepal that demonstrated limited uptake of mental illness detection strategies among community health volunteers [34]. However, the current study differs by focusing on primary care workers and using the app as a decision aid tool, rather than simply documentation and communication.

Another important challenge in using mobile app-based clinical guide reported in the study was the additional burden for healthcare workers for entering patients’ data in both mobile app and paper-based patients register. Considering the high workload of primary health workers in Nepal, they might be less motivated in using mobile app if patients’ data and clinical notes are to be entered in both patients’ domains. If paper forms were to be replaced by electronic forms in the near future, health workers might be motivated to use electronic forms without reservation however this is not likely in the context of Nepal. These results are also supported by a pilot study of m-health application in multidrug-resistant tuberculosis (MDR-TB) program in South Africa, where a very poor uptake of m-health application was reported despite health workers’ positive response towards m-health [49].

Other barriers and risk factors in using mobile app-based clinical guidelines identified in our study were potential distraction in the communication between patients and health workers, and breaking confidentiality of the patients’ data. Some of these challenges can be minimized by training health care workers adequately in the app, using the app only after the initial consultation, and using password protected mobile phones. If the privacy and confidentiality of patient data are not maintained appropriately patients may not be comfortable in sharing their personal information. About half of the study population in the US national survey had concerns about privacy and security of their medical records [50]. Similar results have also been reported in a separate study where 50% patients expressed concerns regarding privacy issues, and 40% felt uncomfortable about providing their personal information to clinicians [51].

The findings of this study may have several implications to improve the reach and coverage, as well as the quality of the mental health services provided through primary health care settings. First, the results demonstrated that most of the health workers use android mobile, and are experienced in using technology in health care. They were highly motivated and positive in using mobile technology in mental health care. This approach, therefore, could be a viable and effective strategy in building clinical capacity of primary health care providers in mental health care in Nepal. The mobile app-based clinical guideline, which is currently being developed, is based on the second version of the mhGAP-IG [22], the first version has already been used in more than 100 countries [52]. The mhGAP-IG based district mental health care plan was also effective in improving treatment coverage, accurate detection of mental disorders and initiation of minimally adequate treatment in primary care, and positive clinical outcomes in Nepal [36].

The mhGAP-IG version 1.0 has also been translated and adapted in Nepal, and hundreds of primary health care workers have already been trained in mhGAP-IG version 1.0. Moreover, the Ministry of Health in Nepal has developed treatment protocols and training manuals based on the mGAP-IG; therefore, the same materials and protocol could also be used in mobile app-based clinical guidelines. This strategic approach could be helpful for scaling up of primary care based mental health treatment throughout the country. Second, the government of Nepal has initiated telemedicine programs in several rural districts with the aim of providing specialists services from rural district hospitals. However, it has been reported that these services were not effective due to lack of information technology infrastructure, and competent technical person to operate the system in the rural hospital [33]. Although, there are no systematic data on cost-effectiveness of telemedicine system [53]; mobile app-based clinical guide may require less technical persons for its day-to-day operation compared to the telemedicine system. Therefore, m-health could be an appropriate and effective strategy to provide basic mental health services in the remote districts where both specialists’ mental health services and technical expertise are not present.

Lastly, our previous studies found that one-time mental health training to primary healthcare workers was insufficient to improve performance [36] and regular supervision and mentoring from the mental health specialists was necessary to see improvement. Our previous project created an innovative approach of supervision, “psychiatric case conference”, where trained health care workers were invited to a specific venue to address their difficulties in assessment, detection and management of mental health disorder [54]. Although, the psychiatric case conferences were found to be effective and were also appreciated by the trained health care workers [37], this approach requires physical presence of both mental health specialists and primary health care workers. One of the features in the app-based clinical guide suggested by the health care workers is collection of patients’ socio-demographic and clinical data which can also be used by a mental health specialist or clinical mentoring and supervision of the trained health workers remotely. Finally, as suggested by health care workers, if the mobile app could include a feature of sending reminders to patients for follow-up visits, this could help to minimize the high dropout rate in mental health care [37]. This is the first study that has explored the perceptions of healthcare workers on feasibility, acceptability and benefits of using a mobile app based clinical guideline for mental health care in primary health care settings. This study will provide valuable insights and information for the development and use of mobile app based clinical guidelines in low resource settings. The study has a few limitations. First, the study was conducted in two districts namely Chitwan and Ramechhap; therefore, the results may not be generalizable to all health workers in Nepal. Second, the study was conducted with prescribing health workers (i.e. health assistants, auxiliary health workers and medical officers); therefore, the results may not represent the opinions of non-prescribing health workers (e.g. nurse and auxiliary nurse midwives).

## Conclusion

This is, to the best of our knowledge, the first study assessing the perceptions of primary health care workers on the feasibility, acceptability and benefits of using mobile app-based clinical guide for detection and treatment of mental disorders in the primary healthcare setting in Nepal. Most of the health workers participated in the study found to be highly interested and motivated in using mobile app-based clinical guides for detection and treatment of mental disorders in primary care. The key features and functions suggested by the health workers in the mobile app-based clinical guides, i.e. suggestive diagnosis and treatment options, patients’ data recording system, messaging, and offline functions, are instrumental to inform the design of the app. The suggested functions and features as well as the potential risk factors highlighted by the health workers, will be considered when further developing the mobile app-based clinical guidelines, training modality and materials, and supervision system.

## Data Availability

Interested parties may notify the EMILIA investigators of their interest in collaboration, including access to the data-set analysed here, through the following email: nicole.votruba@kcl.ac.uk.

## Abbreviations

AHW: Auxiliary health worker
AUD: Alcohol used disorder
EIRS: Electronic immunization reporting system
FGD: Focus group discussion
HA: Health assistant
HCD: Human Centered Design
HMIS: Health Management Information System
e-mhGAP IG: Electronic Mental Health Gap Action Program Intervention Guide
HP: Health post
IMNCI: Integrated Management of Newborn and Childhood Illness
LMICs: Low or middle-income countries
MDR-TB: Multidrug resistant tuberculosis
mhGAP IG: Mental Health Gap Action Program Intervention Guide
MO: Medical officer
OPD: Out patient department
PHCC: Primary Health Care Center
S-AHW: Senior auxiliary health worker
SMS: Short message service
SSI: Semi-structured interviews
WHO: World Health Organization
